# WLS-ILIAD: New Longitudinal Resource for Cognitive and Dementia Data

**DOI:** 10.1093/geroni/igab046.852

**Published:** 2021-12-17

**Authors:** Sanjay Asthana, Pamela Herd, Victoria Williams

**Affiliations:** 1 University of Wisconsin, Madison, Wisconsin, United States; 2 Georgetown University, Georgetown University, District of Columbia, United States; 3 Division of Geriatrics and Gerontology, UW-Madison,, University of Wisconsin-Madison, Wisconsin, United States

## Abstract

One of the distinctive strengths of WLS is the availability of Henmon-Nelson IQ scores on all participants while in high school, followed by prospective collection of data through cognitive batteries of varying size and sophistication. Launched in 1993, the initial longitudinal cognitive testing included 8 abstract reasoning items followed by the administration of larger cognitive batteries in 2004 and 2011 comprised of a 10-item word recall test, digit ordering task, phonemic and category fluency, as well as repeated and new items from the WAIS-R similarities task first administered in the 1993 survey. In 2018, with R01 funding from NIA, the scope of cognitive testing expanded significantly and includes administration of a phone-based cognitive screening measure, and a comprehensive in-person neuropsychological assessment for individuals identified at risk for dementia targeting a range of cognitive domains, including memory, language, attention, visuospatial abilities, and executive functioning.

